# Comparison of Genital *Chlamydia trachomatis* Infection Incidence Between Women With Infertility and Healthy Women in Iran Using PCR and Immunofluorescence Methods

**DOI:** 10.5812/jjm.9450

**Published:** 2014-04-01

**Authors:** Seyed Mahmoud Amin Marashi, Zahra Moulana, Abbas Ali Imani Fooladi, Mohammad Mashhadi Karim

**Affiliations:** 1 Department of Microbiology and Immunology, Alborz University of Medical Sciences, Karaj, IR Iran; 2 Infectious Diseases and Tropical Medicine Research Center, Babol University of Medical Sciences, Babol, IR Iran; 3 Applied Microbiology Research Center, Baqiyatallah University of Medical Sciences, Tehran, IR Iran

**Keywords:** *Chlamydia trachomatis*, Infertility, Women, Iran

## Abstract

**Background:**

For a long time, infertility has been one of the most sequels in medical sciences with microbial agents as one group of its causes. The possible etiological role of *Chlamydia trachomatis* in infertility was suggested years ago, but it has not yet been proved completely. To decrease the severe involvements of *C. trachomatis* infections, screening by efficient diagnostic methods are necessary.

**Objectives:**

In this study we attempted to determine the incidence of *C. trachomatis* in infertile women and compared this with healthy women.

**Materials and Methods:**

This case-control study was performed on 150 infertile women with unknown causes and without physiological deficiency for infertility. The control group consisted of 200 fertile safe and impregnated women. Presence of *C. trachomatis* in the two groups was examined by direct and indirect immunofluorescence tests and PCR.

**Results:**

*C. trachomatis* was detected by direct immunofluorescence method in 23 (15.3%) infertile women compared and 7 (3.5%) healthy controls. Using indirect immunofluorescence tests, a positive test titer of 1:16 as well as the above results were detected in 34 (22.6%) of the infertile cases and 9 (4.5%) of the controls. *C. trachomatis* was detected by PCR method in 48 (32%) infertile women and 13 (8.7%) among the controls.

**Conclusions:**

The results of our study suggest that there is a significant association between *C. trachomatis* infection and female infertility.

## 1. Background

Urogenital infections are caused by *Chlamydia trachomatis* worldwide (1). This bacterium is the most prevalent cause of bacterial sexually transmitted diseases (STD). The prevalence of these infections can be different depending on the country and population type. The prevalence of *C. trachomatis* infection among sexually active women in developing countries is higher than developed countries (2, 3). Annually, about one million *C. trachomatis* infections are reported among sexually active young women in the united states, according to the Center for Disease Control and Prevention (CDC) (4). Most of the infected individuals are asymptomatic; so, the majority of these infections are not detected and treated, and this can lead to pelvic inflammatory diseases, with bacteria reaching the upper genital tract of the infected women (5).

 The etiological relation of pelvic inflammatory diseases (PIDs) with microorganisms is not completely determined; but, the presence of a variety of microorganisms including *C. trachomatis*, *Mycoplasma genitalium*, *Neisseria gonorrhea* and some other microorganisms have been shown in lower and upper genital tracts of women with PID (6-11). Nevertheless, *C. trachomatis* is more suspected than other sexually transmitted diseases to causes symptomatic PID (9, 12, 13). PID can induce disorders such as infertility and ectopic pregnancy. These disorders may be the results of destruction of cilia layer of the fallopian and closure of fallopian tube (14). Therefore, detection and control of *C. trachomatis* infection prevalence is necessary to prevent its related sequels. 

The duration of tubal inflammation and damages resulted from infection are two important factors affecting the efficiency of *Chlamydia* control programs. Therefore, it is important to detect and treat the infection before its development to short-term sequels such as pelvic inflammatory diseases, which in turn can develop into long-term sequels such as tubal factor infertility and ectopic pregnancy (15). In Iran, there are low epidemiological data regarding the prevalence of *C. trachomatis* infection and its consequences and it is clear that having more epidemiological knowledge about genital *C. trachomatis* infection prevalence could be very effective in choosing efficient strategies for screening and treatment of such infections.

## 2. Objectives

The purpose of this research was to determine the prevalence of *C. trachomatis* in healthy women and women with infertility disorder to evaluate the association between *C. trachomatis* infections and infertility in Iran.

## 3. Materials and Methods

### 3.1. Population

This research was a case-control study with 350 female participants. The case group consisted of 150 infertile women, 20 to 40 years, with no recognized physiological deficiency for infertility (unknown cause). The control group consisted of 200 impregnated women, 20 to 40 years, with one or more successful childbirths and without any history of infertility (Table 1). 

**Table 1.** The Mean Age of Participants in the Case and Control Groups

Age Groups	Case Group (n = 150)	Control Group (n = 200)
**20**	31	31
**20 - 24**	23	55
**25 - 29**	80	93
**30 - 34**	10	12
**35 - 39**	4	5
**> 40**	2	4
**Age average**	24.3	25.2

### 3.2. Specimen Collection

Two types of specimens from the both case and control groups were used in this study including endocervical mucosa and blood serums. Sampling from endocervical mucosa was performed using the Pap smear method.

### 3.3. Laboratory Methods

Direct and indirect immunofluorescence methods were used for determining the presence or absence of *C. trachomatis* infection in both case and control groups. For direct testing, endocervical swabs were transferred on clean slides and the slides were incubated at room temperature for drying and fixation. One drop of antichlamydial monoclonal antibody (antibody-online, USA) was added to each slide and they were incubated at 37°C for 10 minutes. Then, the slides were washed with phosphate buffered saline (PBS) and distilled water for 10 and 5 minutes, respectively. Finally, the slides were examined for *C. trachomatis* by fluorescence microscopy (Micros, Austria). 

Using this technique, the elementary bodies are observed as fluorescence green spots. For indirect testing, *C. trachomatis* serotypes D-K, L1-L3 and IOL-207 (TWAR) were used as standard antigens. The serotypes were individually grown in eggs and then placed on specific areas of the slides. A spot on the slide was also specified for the mixture of egg yolk in PBS (PAA, Holland) as a negative control. Serums in dilutions ranging from 1:16 to 1:256 were prepared and individually added to the slides. After 30 minutes of incubation at 37°C, the slides were washed and stained with fluorescence antihuman globulin. Finally, the slides were examined by fluorescence microscopy and the results were recorded based on their color and fluorescence intensity (Table 2). 

**Table 2.** Determination of Positivity or Negativity of the Tests Based on the Fluorescence Intensity and Colors of Slides

Fluorescence Intensity and Color	Positivity or Negativity
**Very shiny green**	+++
**Shiny green**	++
**Green**	+
**Yellowish green**	±
**Orange**	-

### 3.4. PCR Mixture and DNA Amplification

Endocervical swab samples were obtained from the patients. Each swab was placed in a sample collection vial containing the buffer (PBS). Several methods are available for DNA extraction. In the present study, we used the boiling method. We selected one pair of oligonucleotide primers (Cinnagen, Iran) specific for a region of the *C. trachomatis* gene (accession No. AB695165.1), coding for the major outer membrane protein (MOMP). The sequences from 5' to 3' of these oligonucleotide primers are as follows:

Forward: 5'-CCTGTGGGGAATCCTGCTGAA-3'

Revers: 5'-GTCGAAAACAAAGTCACCATAGTA-3'

**Figure 1.**
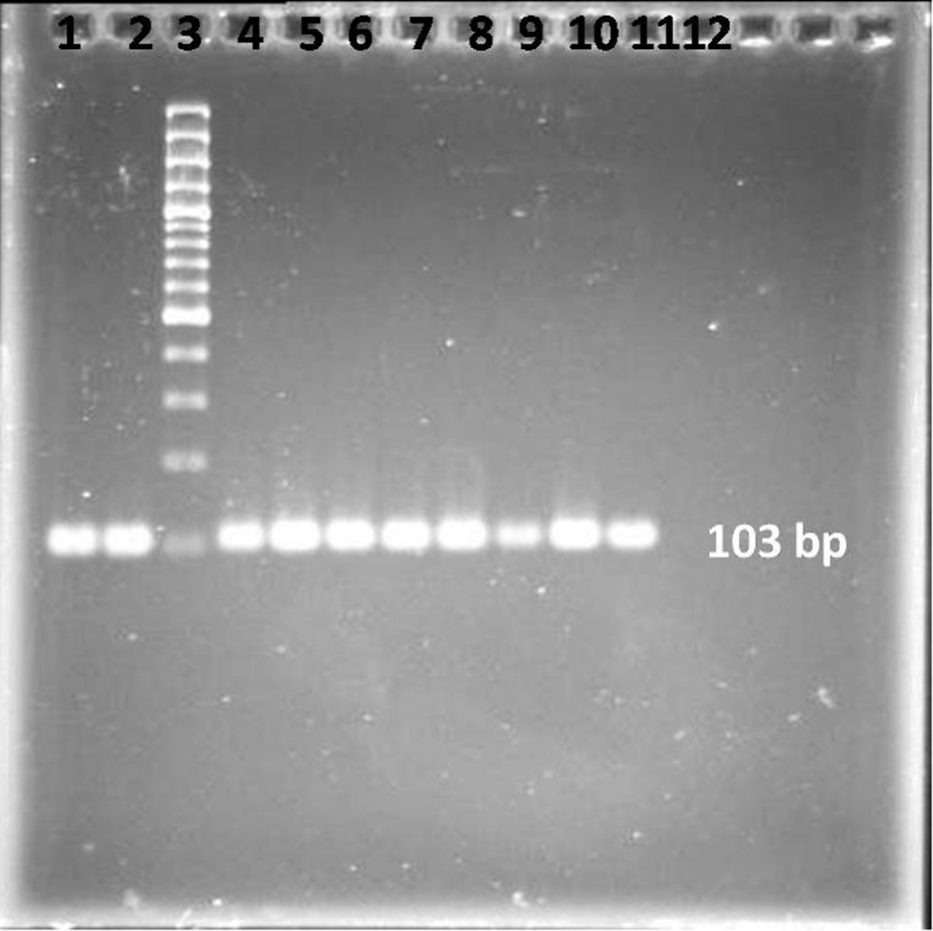
Uniplex PCR for Detection of the MOMP Gene in Clinical Strains of *C. trachomatis* Lanes 1, 2, 4 – 10: clinical samples, lanes 11, 12: positive and negative controls strains respectively, lane 3: molecular weight marker, 100-bp DNA.

A master mixture of these reagents was made for the samples along with the positive and negative controls. The final reaction mixture of 50 µL for each sample contained 0.5 µM of each primer; 100 µM of each of dATP, dCTP, dGTP and dTTP; 50 mM KCl; 10 mM Tris-HCl, pH 8.3; 1.5 mM MgCl_2_, 1.25 units of Taq DNA polymerase enzyme, and 9 µL of the sample DNA. All the PCR reagents were purchased from the SinaClon company, Iran. Each microfuge tube containing the PCR mixture of 50 µL was mixed and subjected to 40 cycles of amplification. Each cycle composed of sequential incubations at 94°C for 1 minute for DNA denaturation, 52°C for 1 minute for annealing the primer to the templates, and 72°C for 30 seconds for DNA chain extension. At the end of the 40 cycles, the samples were kept for another 7 minutes at 72°C for completion of the extension of a DNA chain. The PCR product samples were immediately frozen for further analysis. The PCR products were visualized by agarose gel electrophoresis. A 10-µL post-PCR mixture was subjected to electrophoresis on 2% agarose gel (SinaClone, Iran) in the presence of ethidium bromide (Sigma, Germany). A DNA ladder (SinaClone, Iran) was also run simultaneously to confirm the size of the amplified product (103 bp) (Figure 1). The DNAs were extracted from the bands on the gel using a gel extraction kit (Qiagen, GmbH, Germany) and then sequenced by Macrogen Inc, Seoul, Korea.

## 4. Results

The results of direct and indirect immunofluorescence tests are shown in Table 3. As they show, the prevalence of *C. trachomatis* in the case and control groups reached to 15.3% and 3.5%, respectively, using the direct immunofluorescence method. For the indirect immunofluorescence method, the prevalence of *C. trachomatis* reached to 22.6% and 4.5% in the case and control groups, respectively. In addition, the prevalence of *C. trachomatis* in the case and control groups reached to 32% and 8.7%, respectively, using PCR method. The P values for direct and indirect immunofluorescence and PCR results were calculated by the Fisher test where a P value < 0.001 for direct test results and P < 0.004 for indirect and PCR test results were obtained (Table 3). 

**Table 3.** Results of Direct and Indirect Immunofluorescence and PCR Tests for Diagnosis of *C. trachomatis* in the Case and Control Groups

Anti-Chlamydial Antibody Titer	Case Group Positives (n = 150)	Control Group Positives (n = 200)	Fisher Test Results
**Direct immunofluorescence**	23 (15.3)	7 (3.5)	P < 0.001
**Indirect immunofluorescence**	10 (6.6)	5 (2.5)	
1:16			
1:32	18 (12)	2 (1)	
1:64	6 (4)	2 (1)	
1:128	0	0	
**PCR**	48(32)	13 (8.7)	P < 0.004

## 5. Discussion

*C. trachomatis* is one of the most prevalent causes of sexually transmitted diseases. It is the major cause of urethritis and cervicitis, as well as their sequels such as PID and tubal factor infertility (16). Generally, Chlamydial infections are more destructive for reproductive health of women than men (5).

Svenstrup et al. reported that 23% of women suffering from tubal factor infertility (TFI) had antibodies against *C. trachomatis,* compared with 15% of women in the control group with normal tubes (17). In another research, Siemer et al. showed considerably higher prevalence of IgG and IgA antibodies (39% and 14%, respectively) among women with infertility compared with members of the control group (19% and 3%, respectively) (18). In a study carried out by Malik et al. the presence of *C. trachomatis* in infertile women was 28.1%, which was significantly higher than that of healthy women (3.3%) (P < 0.01) (19). Gaudoin et al. reported that 91.2% of woman with tubal occlusion had IgG antichlamydial antibodies in their sera (20).

Our results were compatible with the above studies.

In our study, the presence of *C. trachomatis* infection in the 150 infertile women with an average age of 24.3 years as the case group as well as 200 healthy women with an average age of 25.2 years as the control group was examined using direct and indirect immunofluorescence and PCR methods. In the direct immunofluorescence method, 15.3% of the case and 3.5% of the control group members showed positive results. The indirect immunofluorescence test also determined that 22.6% of the case and 4.5% of the control group members had a titer ≥ 1:16. The obtained P values from the results of direct and indirect immunofluorescence experiments by Fisher test were P < 0.001 and P < 0.004, respectively, confirming the significant association between urogenital chlamydial infections and infertility. The difference between the results of direct and indirect immunofluorescence tests could be due to old chlamydial infections or infections in other areas of the body.

Our results suggested that there was a significant association between *C. trachomatis* infection and female infertility. Therefore, *C. trachomatis* can be one of the main ethological factors for female infertility. Consequently, it is recommended that infertile women without any physiological deficiency should be examined for contamination with *C. trachomatis*. Finally, for detection of genital *C. trachomatis*, PCR results are more reliable than immunofluorescence tests.

## References

[1] Genuis SJ, Genuis SK (2004). Managing the sexually transmitted disease pandemic: a time for reevaluation.. Am J Obstet Gynecol..

[2] Gerbase AC, Rowley JT, Heymann DH, Berkley SF, Piot P (1998). Global prevalence and incidence estimates of selected curable STDs.. Sex Transm Infect..

[3] Biendo M, Lefebvre JF, Fuentes V, Orfila J (1994). [The prevalence of anti-Chlamydia trachomatis and anti-Chlamydia pneumoniae antibodies in Brazzaville].. Bull Soc Pathol Exot..

[4] Essig A, Murray P. R, Jorgensen J. H, Landry M. L, Pfaller M. A (2007). Chlamydia and chlamydophila.. Manual of Clinical Microbiology..

[5] Peipert JF (2003). Clinical practice. Genital chlamydial infections.. N Engl J Med..

[6] Haggerty CL, Hillier SL, Bass DC, Ness RB, P. I. D. Evaluation, Clinical Health study I (2004). Bacterial vaginosis and anaerobic bacteria are associated with endometritis.. Clin Infect Dis..

[7] Haggerty CL (2008). Evidence for a role of Mycoplasma genitalium in pelvic inflammatory disease.. Curr Opin Infect Dis..

[8] Hillier SL, Kiviat NB, Hawes SE, Hasselquist MB, Hanssen PW, Eschenbach DA (1996). Role of bacterial vaginosis-associated microorganisms in endometritis.. Am J Obstet Gynecol..

[9] Ness RB, Soper DE, Holley RL, Peipert J, Randall H, Sweet RL (2002). Effectiveness of inpatient and outpatient treatment strategies for women with pelvic inflammatory disease: results from the Pelvic Inflammatory Disease Evaluation and Clinical Health (PEACH) Randomized Trial.. Am J Obstet Gynecol..

[10] Ness RB, Kip KE, Hillier SL, Soper DE, Stamm CA, Sweet RL (2005). A cluster analysis of bacterial vaginosis-associated microflora and pelvic inflammatory disease.. Am J Epidemiol..

[11] Simms I, Eastick K, Mallinson H, Thomas K, Gokhale R, Hay P (2003). Associations between Mycoplasma genitalium, Chlamydia trachomatis and pelvic inflammatory disease.. J Clin Pathol..

[12] Heinonen PK, Miettinen A (1994). Laparoscopic study on the microbiology and severity of acute pelvic inflammatory disease.. Eur J Obstet Gynecol Reprod Biol..

[13] Ness RB, Randall H, Richter HE, Peipert JF, Montagno A, Soper DE (2004). Condom use and the risk of recurrent pelvic inflammatory disease, chronic pelvic pain, or infertility following an episode of pelvic inflammatory disease.. Am J Public Health..

[14] Haggerty CL, Gottlieb SL, Taylor BD, Low N, Xu F, Ness RB (2010). Risk of sequelae after Chlamydia trachomatis genital infection in women.. J Infect Dis..

[15] Stamm W, Holmes K, Sparling P, WE S (2008). Chlamydia trachomatis infections in the adult.. Sexually transmitted diseases..

[16] Millman K, Black CM, Johnson RE, Stamm WE, Jones RB, Hook EW (2004). Population-based genetic and evolutionary analysis of Chlamydia trachomatis urogenital strain variation in the United States.. J Bacteriol..

[17] Svenstrup HF, Fedder J, Kristoffersen SE, Trolle B, Birkelund S, Christiansen G (2008). Mycoplasma genitalium, Chlamydia trachomatis, and tubal factor infertility--a prospective study.. Fertil Steril..

[18] Siemer J, Theile O, Larbi Y, Fasching PA, Danso KA, Kreienberg R (2008). Chlamydia trachomatis infection as a risk factor for infertility among women in Ghana, West Africa.. Am J Trop Med Hyg..

[19] Malik A, Jain S, Hakim S, Shukla I, Rizvi M (2006). Chlamydia trachomatis infection & female infertility.. Indian J Med Res..

[20] Gaudoin M, Rekha P, Morris A, Lynch J, Acharya U (1999). Bacterial vaginosis and past chlamydial infection are strongly and independently associated with tubal infertility but do not affect in vitro fertilization success rates.. Fertil Steril..

